# Commentary: Efficacy, safety, and therapeutic drug monitoring of polymyxin B sulfate and colistin sulfate in critically ill patients: a real-world retrospective study

**DOI:** 10.3389/fphar.2026.1757787

**Published:** 2026-03-25

**Authors:** Genzhu Wang

**Affiliations:** Department of Clinical Pharmacy, Beijing Electric Power Hospital of State Grid Co. of China, Capital Medical University Electric Teaching Hospital, Beijing, China

**Keywords:** acute kidney injury, clinical efficacy, colistin sulfate, polymyxin B, therapeutic drug monitoring

## Introduction

Multidrug-resistant Gram-negative bacteria (MDR-GNB) pose a severe threat to global public health, particularly in critically ill patient populations, where they are associated with high mortality and morbidity (Cassini et al., 2019; Leone et al., 2023). Polymyxin B sulfate (PBS) and colistin sulfate (CS) remain important antimicrobial agents for managing MDR-GNB infections in clinical practice, especially in regions with limited access to novel anti-infective drugs (Infectious Diseases Society of China et al., 2021). Given their narrow therapeutic window and inherent nephrotoxicity, therapeutic drug monitoring (TDM) has become a key clinical strategy to optimize polymyxin dosing and balance efficacy and safety (Tsuji et al., 2019; Abdul-Aziz et al., 2020).

Zhang et al. recently published a valuable real-world retrospective study that compared the efficacy, safety, and TDM applications of PBS and CS in 180 critically ill patients with MDR-GNB infections (Zhang et al., 2025). The study employed propensity score matching (PSM) to balance baseline characteristics between the PBS (n = 100) and CS (n = 80) cohorts and identified no significant differences in clinical efficacy, 30-day mortality, or overall acute kidney injury (AKI) incidence between the two agents. Notably, the study found a higher incidence of stage 3 AKI in the PBS cohort and established TDM trough concentration (C_min_) thresholds for clinical efficacy: ≥0.91 mg/L for PBS and ≥0.53 mg/L for CS. Additionally, the authors’ conclusion that TDM significantly improves clinical efficacy and reduces AKI risk for both drugs is a clinically meaningful finding.

This single-center retrospective study provides some useful data on polymyxin clinical use. However, the findings raise several questions regarding their interpretation and highlight potential uncertainties in the methodology, presentation of TDM and pharmacokinetic data. These uncertainties require further clarification to refine our understanding of polymyxin TDM in critically ill patients and guide future clinical research and practice.

## Interpreting the impact of TDM

In the abstract, the authors’ conclude that TDM significantly improves clinical efficacy and reduces acute kidney injury (AKI) incidence for both drugs—a clinically meaningful finding that aligns with international consensus guidelines on polymyxin use (Tsuji et al., 2019; Infectious Diseases Society of China et al., 2021). However, several details in the study design and data presentation create uncertainties in interpreting the causal relationship between TDM implementation and treatment outcomes, which require further contextualization.

First, the study does not report whether and how drug regimens were adjusted based on TDM results. The clinical value of TDM relies on using drug concentrations to guide dose adjustments. However, because this study lacks data on specific dose modifications and their timing, it is difficult to assess TDM’s true impact on treatment outcomes. Second, potential selection bias could confound the association between TDM and improved outcomes, as physicians who routinely order TDM may also provide more intensive clinical monitoring and supportive care for patients. Clarifying the TDM-guided dosing adjustment strategies used in the study would help isolate the true effect of drug monitoring on treatment efficacy and safety.

In the methods section, the study reports off-label high-dose CS use (1.0 million units q12h), which exceeds the maximum dosage specified in the Chinese product insert. While off-label dosing is common in critically ill patients with MDR-GNB infections to achieve therapeutic concentrations, the study does not contextualize the rationale for this dosing strategy—e.g., whether it was based on TDM results, disease severity, bacterial resistance patterns, or other factors. The relationship between this off-label dosing and TDM implementation remains unclear, and exploring this association would provide valuable insights into the clinical utility of TDM for guiding high-dose CS use in critically ill patients.

A notable finding is the significant difference in TDM practice rates between the PBS and CS cohorts, both before (70.0% vs. 42.5%, p = 0.001) and after (81.4% vs. 55.9%, p = 0.003) propensity score matching (Table 1 of the original article). Given the study’s conclusion that TDM enhances clinical efficacy and reduces AKI incidence, the absence of a corresponding difference in these clinical outcomes between the two cohorts is an unexpected observation that points to potential unmeasured confounding factors. These factors may include variations in the timing of TDM sampling or differences in concomitant supportive care between the two groups, all of which could mask the expected relationship between TDM rate and treatment outcomes. Further exploration of these confounding factors in subgroup analyses would help refine the interpretation of TDM’s impact.

**TABLE 1:** Resistance rates of PBS and CS against Gram-negative bacteria.

Type of Gram-negative bacteria	Resistance rate (%)	
	PBS	CS
*Acinetobacter baumannii*	1.8	1.8
Carbapenem resistant *A. baumannii*	1.1	1.1
*Klebsiella pneumoniae*	6.7	4.1
Carbapenem resistant *K. pneumoniae*	10.9	11.8
*Pseudomonas aeruginosa*	1.2	2.3
Carbapenem resistant *P. aeruginosa*	2	6
*Escherichia coli*	1.3	1
Carbapenem resistant *E. coli*	NA	NA

Abbreviations: PBS, Polymyxin B sulfate; CS, colistin sulfate; MIC, minimum inhibitory concentration; NA, not available.

## The C_min_ thresholds and clinical efficacy

A novel finding of this study is the identification of the distinct C_min_ efficacy thresholds (≥0.91 mg/L for PBS and ≥ 0.53 mg/L for CS), which offers practical guidance for TDM in Chinese critically ill patients. However, this finding warrants further discussion in relation to other studies in the field. Current research shows similar minimum inhibitory concentration (MIC) values for both drugs against MDR-GNB (Chew et al., 2017; Behera et al., 2018; Yildiz et al., 2021). In addition, the Clinical and Laboratory Standards Institute (CLSI) has indicated that MICs obtained from testing CS predict MICs to PBS and *vice versa* (CLSI, 2020), demonstrating the cross-susceptibility expected of agents within the same class. The Chinese Antimicrobial Surveillance Network (CHINET) (2023) demonstrates comparable resistance rates against Gram-negative bacteria, with PBS demonstrating slightly higher *in vitro* sensitivity than CS (Fupin et al., 2023), as shown in Table 1. Additionally, recent research has found that PBS and CS exhibit similar synergistic effects when combined with common clinical antimicrobials against MDR-GNB, with PBS showing marginally better combined efficacy (Wang et al., 2024).

Based on these findings, CS achieves clinical efficacy at a nearly 50% lower C_min_ than PBS, which is difficult to reconcile with existing evidence. Two potential factors may explain this discrepancy: 1. the study did not report MIC values of the isolated MDR-GNB strains to PBS and/or CS, making it impossible to correlate the established C_min_ thresholds with *in vitro* susceptibility data; 2. the small sample size of TDM data (70 PBS patients, 34 CS patients) may limit the generalizability of the established thresholds. Future research combining *in vitro* MIC data with *in vivo* TDM concentrations is needed to validate these C_min_ thresholds and explore their relationship with antimicrobial susceptibility.

Notably, the study states that the C_min_ values of PBS and CS are significantly correlated with the clinical efficacy and AKI incidence, a key finding for understanding the polymyxin therapeutic window. However, these correlation data are not presented in the Results section. Presenting this data would provide a more comprehensive understanding of the trade-off between efficacy and nephrotoxicity at different polymyxin concentrations, a critical consideration for TDM-guided dosing.

## Stage 3 AKI in PBS and CS cohorts

The finding of a significantly higher incidence of stage 3 AKI in the PBS cohort (11.0% vs. 2.5% before PSM; 10.2% vs. 3.4% after PSM) is a clinically important and consistent with recent studies (Zhang et al., 2024). The Kaplan-Meier curve for stage 3 AKI (Figure 3 of the original article) further shows a notable difference: stage 3 AKI in the CS cohort occurs only in the early treatment phase, while it increases over time in the PBS cohort, suggesting potential drug accumulation in the PBS group. This result raises several pharmacokinetic and dose-related questions. First, the study reports no significant difference in baseline creatinine clearance between the PBS and CS cohorts, a key factor influencing polymyxin elimination (Fiaccadori et al., 2016; Xie et al., 2022; Fang et al., 2024). In healthy populations, PBS and CS have reported half-lives of 5.44 h and 4 h, respectively (Fang et al., 2024; Huang et al., 2024)—a small but potentially meaningful difference that could lead to cumulative drug exposure in critically ill patients with impaired renal function. However, the study does not report serial C_min_ measurements in patients with multiple TDM samplings, particularly before and after 7 days of treatment. Analyzing these longitudinal concentration data would help determine whether the increased late-stage 3 AKI in the PBS cohort is associated with rising drug concentrations. Second, the study does not present detailed data on the dosing frequency of PBS and CS in the two cohorts, a factor that can influence drug accumulation and nephrotoxicity. While the study lists the common dosing regimens for both agents, it does not report the distribution of dosing frequencies (e.g., q8h vs. q12h) in the PBS and CS groups. Given PBS’s longer half-life, more frequent dosing in the PBS cohort could contribute to cumulative exposure and late-stage AKI, and exploring this association would provide valuable insights into the impact of cumulative exposure on polymyxin nephrotoxicity. It is important to note that the absolute number of stage 3 AKI cases in both cohorts is small (11 in PBS, 2 in CS before PSM), which limits the statistical power to identify the exact causes of the observed difference. Additionally, MDR-GNB infections in critically ill patients are almost always treated with combination antimicrobial therapy, and the study reports concomitant use of other nephrotoxic agents (e.g., amikacin, sulfamethoxazole/trimethoprim) in both cohorts. While the study controls for some confounding factors via PSM, the potential synergistic nephrotoxicity of polymyxin-combination therapy remains an unmeasured factor that may influence the observed AKI rates. Future large-sample studies are needed to explore the interaction between polymyxin type, dosing frequency, cumulative exposure, and concomitant nephrotoxic drugs on AKI risk in critically ill patients.

## Polymyxin clinical use and formulation distinctions

The original study states that PBS and CS are the last-line treatments for MDR-GNB infections due to a lack of more effective alternatives. However, newer β-lactam/β-lactamase inhibitor combinations (e.g., ceftazidime-avibactam, imipenem-relebactam, aztreonam-avibactam, ceftolozane-tazobactam, among others) have demonstrated superior efficacy and safety to polymyxins and are now recommended as first-line options in many settings for CRKP and CRPA infections (Paul et al., 2022; Pintado et al., 2023). Similarly, for CRAB infections, sulbactam-based strategies have emerged as first-line options in many settings (Zeng et al., 2023). Polymyxins remain important salvage therapies for MDR-GNB infections, particularly in regions with limited access to novel antimicrobials.

The original study notes that CS is only available in China but does not clarify the critical pharmacological and clinical distinction between CS and colistin methanesulfonate sodium (CMS)—the internationally more common colistin formulation. They have similar chemical structures and mechanisms of action, but their pharmacokinetics, dosing, and nephrotoxicity differ. CMS is a prodrug that is converted to the active moiety colistin *in vivo,* whereas CS is administered in its active form (Fiaccadori et al., 2016; Xie et al., 2022). CMS is mainly eliminated by the kidneys, while CS is primarily excreted through non-renal pathways (Tran et al., 2016). The conversion factor is 1 million IU, equivalent to approximately 33 mg CBA. Dose adjustment is recommended for CMS in patients with decreased creatinine clearance, but not for CS (Tsuji et al., 2019; Zhang et al., 2024). Nephrotoxicity is the most common and prominent adverse effect of polymyxins. The reported rates of nephrotoxicity associated with CMS and CS vary extensively in previous studies (Yang et al., 2025; Zeng et al., 2025). As reported in previous studies, the incidence of acute kidney injury is 15.6%–59.3% for CMS (Rosas Espinoza et al., 2021; Simon et al., 2023; Yang et al., 2025) and 5.9%–28.8% for CS (Liu et al., 2024; Zhang et al., 2024; Zeng et al., 2025; Zhang et al., 2025).

In conclusion, Zhang et al.’s study is a valuable contribution to polymyxins research, and addressing the identified uncertainties will further strengthen the value of its findings for clinical practice and future research. As polymyxins remain core agents for MDR-GNB infections in China as well as in other countries, real-world studies combining TDM data with pharmacokinetic, microbiological, and clinical outcomes will be critical to optimizing their use and balancing efficacy and safety in critically ill patients.
